# Experiences of deaf women and girls in accessing maternal health rights and services in Uganda

**DOI:** 10.4102/ajod.v14i0.1627

**Published:** 2025-07-16

**Authors:** Esther M.A. Gimono

**Affiliations:** 1 Department of Philosophy, School of Liberal and Performing Arts, Makerere University, Kampala, Uganda

**Keywords:** maternal health rights and services, deaf women, deaf girls, sign language, Ugandan sign language, Uganda, Mbale district

## Abstract

**Background:**

Women with disabilities are at disproportionate risk for adverse pregnancy outcomes partly because of the limited information on their pregnancy histories. However, deaf women are faced with communication challenges, sexuality, menstrual health as well as pregnancy and its care, which remain a contemporary phenomenon. Still, little is known about the lived experiences of deaf women and girls.

**Objectives:**

The aim of this study was to examine the maternal health experiences of deaf women and girls, identify the challenges that influence their antenatal, childbirth and postnatal outcomes and improve access.

**Method:**

The study used qualitative research of an intrinsic case study design utilising semi-structured interviews and focus group discussions with 50 deaf women and girls who are deaf or hard of hearing in Mbale district and 13 key informants from state and non-state entities. Documentary analysis was also utilised to examine government documents on this topic.

**Results:**

Findings revealed that 100% of deaf women and girls lack antenatal services tailored to their linguistic needs and communication barriers, which provide no opportunities for better medical provider–patient communication.

**Conclusion:**

Despite Uganda’s legal frameworks on maternal health rights (MHRs), deaf women and girls’ linguistic needs are yet to be incorporated into the Ugandan health sector. Current healthcare provisions do not always meet their needs during maternal services. Therefore, visible and constructive policies are necessary to steer deaf MHRs and services.

**Contribution:**

Deaf epistemology should be integrated into policy, research spaces and practice for effective and evidence-based policies needed to guide Sexual and Reproductive Health services among deaf women and girls.

## Introduction

Women with disabilities are at disproportionate risk for adverse pregnancy outcomes partly because of the limited information on their pregnancy histories (Gichane et al. 2017). They face particular barriers to their rights, as gender and disability intersect (Al-Nashif 2020). There are widely held beliefs that the bodies of persons with disabilities (PWDs) in general and women with disabilities in particular are ugly, shameful and unattractive (Twinomugisha 2018). Studies that have explored the maternal healthcare experiences of women with disabilities have found that they face a myriad of challenges in accessing and receiving care (Redshaw et al. 2013; Twinomugisha 2018). Although different empirical studies have been done on maternal healthcare of women with disabilities (Ganle et al. 2016; Gichane et al. 2017; Twinomugisha 2018), including deaf women, little is known about the lived experiences of maternal health rights (MHR) and services in Uganda.

Deaf women are members of a minority group faced with communication challenges, especially where verbal communication is needed (Adigun, Akinrinoye & Obilor 2021). Thus, the inability to actively communicate and interact adequately in situations where oral communication is required has put the deaf at a disadvantage (Adigun et al. 2021; Mitra et al. 2020). Among the challenging issues related to deaf women’s health are sexual and menstrual health, as well as pregnancy and its care, which remain a contemporary phenomenon (Adigun & Mngomezulu 2020). In particular, pregnancy care for deaf pregnant women vis-à-vis antenatal registration, visits and associated care is still unclear (Adigun & Mngomezulu 2020). Scholars like Mitra et al. (2020) revealed that deaf and hard-of-hearing women are more likely to have chronic diseases, pregnancy complications and poor birth outcomes than hearing women.

Available data suggest that when it comes to the experience of maternity services, deaf women report lower satisfaction with the overall quality of antenatal care, fewer antenatal appointments and poor communication with physicians, but when provided with access to the interpretation services they need, they report higher satisfaction (Adigun et al. 2021; Gichane et al. 2017; O’Hearn 2006). Additionally, evidence further suggests that communication barriers contribute to lower utilisation of health services by deaf women (Gichane et al. 2017). And they are more likely to have fewer prenatal visits and are frequently less satisfied with the prenatal care they receive than their hearing counterparts (O’Hearn 2006).

The rights of PWDs are provided in the Convention on the Rights of Persons with Disabilities, 2008 CRPD (2008). Uganda is a state party to this convention, which obliges the government to respect, fulfil and protect the rights of PWDs based on the human rights principles of equality, non-discrimination, effective participation and inclusion in communities; respect for inherent dignity and autonomy; equality between women and men and respect for the evolving rights of children with disabilities (NUDPU 2011).

The CRPD provides for the rights of women with disabilities, including Maternal Health Rights under Article 6(1), which provides that State Parties recognise that women and girls with disabilities are subject to multiple discrimination and, in this regard, shall take measures to ensure the full and equal enjoyment by them of all human rights and fundamental freedoms (CRPD 2008). Article 6(2) further shows that State Parties shall take all appropriate measures to ensure the full development, advancement and empowerment of women for the purpose of guaranteeing them the exercise and enjoyment of the human rights and fundamental freedoms set out in the convention (CRPD 2008).

The Maputo Protocol, 2003, under Article 23, focuses on the special protection of women with disabilities, emphasising their rights to freedom from violence and discrimination and the right to be treated with dignity (Maputo Protocol 2003). At the national level, the Ugandan Constitution, 1995 as amended provides Article 35, which guarantees the rights of PWDs, stating they have a right to respect and human dignity, and the state and society must ensure they realise their full potential (Constitution of the Republic of Uganda 1995). This Constitution under Article 33(1) guarantees that ‘women shall be accorded full and equal dignity as men’. Moreover, Article 33(3) further recognises the ‘unique status and natural maternal functions of women in society’ (Constitution of the Republic of Uganda 1995). Despite all these legal frameworks, deaf women and girls’ maternal healthcare services are ignored and left out.

According to WHO, maternal health refers to the health of women during pregnancy, childbirth and the postpartum period (Twinomugisha 2017; WHO 2012).

### Theoretical framework

This study used the social model of disability and human rights-based approach (HRBA), which shaped and provided a lens through which the research was analysed. The social model of disability explains disability as a social construct through discrimination and oppression (Degner 2014). Through the social model, disability is considered secondary to the individual (Shakespeare 2010). Instead, the individual’s strengths, aspirations and needs are primary to disability (Shakespeare 2010). This model focuses on barriers and structures that affect the individuals because of their disability (Shakespeare 2010).

While the social model merely explains disability, the HRBA encompasses the values for disability policy that acknowledges the human dignity of disabled persons (Degner 2014). A HRBA aims to increase the capacity of both the duty-bearers and the rights-holders (UNDP 2006; UNFPA 2014). This approach applies the AAAQ framework (availability, accessibility, acceptability and quality), which depends on the conditions prevailing in a particular state party (Rukooko 2012).

This study was to examine MHRs and services among deaf women and girls in the Mbale district. The aim was to gain insights into maternal health awareness and practices to illuminate and redirect future action policies and programmes. As such, this study responded to the following questions: (1) *What are the maternal health rights and services awareness and practices that exist among deaf women and girls?* (2) *What are your sources of information about maternal health rights and services?* (3) *What contributions has the government as a duty bearer made towards implementing maternal health rights and services for deaf women and girls?*

## Research methods and design

This study used an intrinsic case study that focused on deep understanding of deaf women and girls’ unique linguistic needs on access to MHRs and services, which was described and detailed (Creswell 2013). Using qualitative methods, the lived experiences of MHRs and services were examined by means of semi-structured interviews, focus group discussions (FGDs) and documentary analysis, which entailed reviewing or evaluating government documents, both printed and electronic material, on this topic (Bowen 2009).

This study used deaf epistemology that relied heavily on personal testimonies, experiences and personal accounts to document knowledge (Holcomb 2010). As Ladd (2003:19) suggests, deaf epistemology is an opportunity for people to understand clearly ‘deaf ways of being in the world, of conceiving that world and their place within it, both in actuality and in potentiality’. Deaf epistemology provides first-hand accounts on which knowledge is based (Holcomb 2010). This paradigm allowed the participants to freely describe their own experiences and testimonies on MHRs and services and understand their world and develop their particular meanings that correspond to their experience. As a researcher, my role was to listen carefully to their views and interpret the findings based on their background and experiences (Creswell 2013). The interpretation of their experiences revealed a significant amount of information regarding the challenges and new insight into the overall study.

In a nutshell, epistemology addresses the question, *how do we know what we know?* In the case of the deaf world and the field of deaf education, what constitutes true beliefs or justified beliefs? One goal of epistemology is to determine the criteria for knowledge so that we can know what can or cannot be known (Holcomb 2010). This study was carried out in Mbale district in eastern Uganda because Mbale suffered teenage pregnancies, which were shockingly high in 2018, 2019 and 2020 (CEDAW 2022; FAWE 2020; Mukaaya & Chemitai 2021; New Vision 2019; UBOS & ICF 2018; Uganda Bureau of Statistics 2017). Additionally, it is where the largest mixed government-owned secondary school for the deaf is found (Mbale School for the Deaf). Moreover, the study also purposely selected Kampala, where the offices of the key informants are located.

### Research settings

#### Population and sampling techniques

A total of 63 study participants were recruited in this study. The study purposively used 50 deaf women and girls who were focus group participants, and, among these participants, some of them requested for follow-up in-depth interviews. The 13 key informants included three executives from government departments, for instance, Ministry of Health; Ministry of Gender, Labour and Social Development; and Ministry of Education and Sports; 1 executive from the Uganda National Association for the Deaf (UNAD); 2 village health teams (VHTs); 1 school administrator; 2 health workers; 1 community development officer (CDO); 2 sign language interpreters and 1 deaf husband.

The research team contacted the deaf school about the population size of the female deaf students between 15 and 17 years. However, the school declined because of the ongoing court investigation of corruption charges. Moreover, the research team also approached Mbale district headquarters to access the documents of the population size of deaf women in this area. Unfortunately, data were unavailable. Therefore, the research team had to change the recommendations and principles to select the sample size.

Sample size in qualitative research has been the subject of enduring discussions (Morse 1995, 2000; Sandelowski 1995). Despite these discussions, various conceptual developments have sought to address this issue, with guidance and principles and, more recently, an evidence-based approach to sample size determination seeking to ground the discussion empirically and recommend different sample sizes in the process of obtaining data saturation (Lincoln & Guba 1985; Morse 1995, 2000; Sandelowski 1995). For instance, Morse (1995) recommends that phenomenologies and case studies directed towards the essence of experiences include 30–50 participants to obtain saturation in qualitative studies. Sandelowski (1995) reveals that qualitative studies may involve over 50 sample sizes. Moreover, one of the most acceptable standards in qualitative research is to allow the data to reach data saturation (Creswell 2013; Creswell & Creswell 2018).

In terms of principles, Lincoln and Guba (1985) proposed that sample size determination be guided by the criterion of *informational redundancy*; that is, sampling can be terminated when no new information is elicited by sampling more units (Lincoln & Guba 1985). It is upon the above recommendations and principles that this study selected a sample size of 50 participants. Prior to data collection, team and I selected 30 women from Busamaga, then 6 FGDs were formed consisting of 5 participants in each group, but the study applied the principles of informational redundancy as cited in Lincoln and Guba (1985). Then, after a discussion with the 5th FGD, enough data were collected from the participants, and they fully responded to all relevant themes and perspectives, which led to the termination of the process. This means that one group was dropped because it was unlikely to provide significant new information. This led to 25 participants who were utilised. Recruitment was done at the church and Busamaga health centre during weekends.

Similarly, in Busoba village, prior to data collection, the team and I selected 40 girls, and then 8 FGDs were formed, consisting of 5 participants in each group, because of the principles of informational redundancy, as cited in Lincoln and Guba (1985). After a discussion with the 5th FGD, we reached data saturation, and the rest of the 3 groups were dropped because they were unlikely to provide significant new information, which led to 25 participants who were utilised. Therefore, the overall sample size selection was 50 participants. The intent in selecting these villages was to sample participants with diverse characteristics so that views from deaf women and girls with different perspectives on the topic can be reflected.

Firstly, these villages were selected because Busamaga village has the largest referral health centre that offers medical services to more than 24 villages and a community deaf church. Secondly, Busoba village is where the deaf school is located.

#### Inclusion criterion

The criterion for inclusion was based on profound deaf or hard of hearing who only use sign language and had knowledge of MHRs and services and an age range between 15 and 49 years. Therefore, any participant who was not in that age range and the above criterion was excluded. During the selection of participants, the research team met with the local council members of these areas, church leaders, school administrators, health workers, including VHT, and the CDO for the introduction to a PhD study. Then, we were allowed to access all the sites and participants in these areas. Permission was both written and verbal. Deaf women were mobilised through community engagements with VHT, church leaders and the local leaders. The VHT and church leaders were key in identifying the deaf women because they interact with them in health-integrated programmes and church services. In Busamaga village, the team of some local leaders, together with the research team, moved door to door and attended church services to inform the participants about the study. Interestingly, most of the deaf women were delightful for recruitment. They were informed of their ethical rights by disclosing details about the purpose of the study, procedures, potential risks and benefits, allowing them to freely choose to participate without coercion or misunderstanding the details of the study. Later on, they consented to and assented to the interviews and FGDs.

Meanwhile, deaf girls were recruited from Busoba village in Mbale School for the Deaf; permission to carry out interviews and FGDs was sought from the school administration and parents. Consent and assent permission were sought from the girls, parents and the administration of the school because, according to the *Uganda National Council for Science and Technology (UNCST) Act of 2000*, Cap 209, a 17-year-old is considered a minor and would typically need both their own consent and parental consent to participate in any research study (UNCST 2000). Students who participated in the study were met on weekends, and only those who passed the inclusion criterion attended the screening, recruitment, interviews and FGD selection and were considered. While at school, the administrator gave us privacy and we promised the student and staff confidentiality in all discussions.

The study purposively selected three government ministries because during the Second Global Disability Summit (2022) held on 16 – 17 February, the Disability Summit Commitments for the Republic of Uganda were based on the five core themes placed under these three ministries as follows: Under theme 1 and theme 4: Capacity enhancement of PWDs and their organisations. The Government of Uganda, through the three ministries, will facilitate capacity building for PWDs through the organisations and mobilise partnerships for research to inform evidence-based investments in PWDs.

Further, selection of key informants was done by presenting my introduction letter and research ethics committee documents to the offices of permanent secretaries of the targeted government institutions and other stakeholders who are working under disability to inform the study objectives. Then the study was approved and provided with administrative clearance to access these departments. The clearance letter indicated the office and each key informant for the interview. The interviews focused on the contributions of the government as a duty-bearer in promoting MHRs and services among deaf women and girls, as well as how these programmes are implemented in the deaf community.

The study also applied snowball sampling because after interviewing the first key informant, they led the study to another key informant, who dodged the appointment, but referred to another key informant, whom I interviewed. These informants then acted as ‘seeds’ that led the study to their counterparts in a process of snowball sampling. I later discovered that most of the key informants did not have any information on deaf people, and that is why they referred to different officers.

Notably, the deaf husband was purposely selected because he was and still is a support system to his uneducated deaf wife who gave birth to two children, so he escorted and supported his wife during antenatal and childbirth processes. The wife requested a follow-up interview with her husband to also inform the study.

Two female sign language interpreters were also identified and recruited based on knowledge of Ugandan Sign Language (UGSL) and working experience in the deaf community to inform the study. Additionally, sign interpreters used key informants’ protocols. The ability to communicate in sign language was a priority because it is the native language for the deaf. This study was carried out from September 2023 to January 2024. It should be noted that after the data collection process, all the participants were remunerated for their time and transport refund per UNCST Act, 2000 Cap 209.

#### Data collection and analysis

The FGDs and interviews were composed of both semi-structured and open-ended questions regarding awareness and practices of maternal health services (antenatal, post-natal and childbirth). The study utilised 10 FGDs, consisting of 5 participants in each group, which lasted for 1 h. However, among the 50 women and girls, 8 participants requested for follow-up in-depth interviews because of the sensitive information that they withheld during the FGDs. Among these participants were 2 deaf girls and 6 deaf women, and the interview lasted for 45 min per person.

During the interviews, key informants opted for interviews, because of their busy schedules, in their offices. The key informants were also given the consent form for review in advance before meeting for an interview. At all times, the study maintained the participants’ privacy and confidentiality and ensured that all participants knew that they were allowed to withdraw if they chose to do so. It was important to explain to the key informants and participants the research and its expectations to seek their consent before pursuing the interviews and FGDs of collecting data.

Video tapes were used with participants’ permission to record discussions in the focus group. All sessions were conducted by female research assistants. I also helped the assistants when technical or terminological issues were needed for clarification. The research team members were all native signers, so all focus group sessions were conducted in Sign Language (SL). Additionally, we used ‘simultaneous method’ in sign language, which required conveying information by speaking and signing at the same time, using both spoken language and sign language simultaneously to communicate with the participants (Lutalo-Kiingi 2014). Being native signers and members of the deaf community in Uganda made it easy to create a mutual understanding with the participants and created a comfortable environment to share their experiences on MHRs and services.

Transcription of the data from the hard disk drive was done in two steps: partial transcription and full transcription. The first step, partial transcription, involved viewing the videos from all the focus groups and interviews to identify and transcribe information that had been raised by participants and respondents. The second step was a full transcription of the videos. I extensively reviewed the video discussions and field notes. As concepts and themes emerged, coding and organising data to identify different themes and their relationships between them were developed and applied on the transcript.

#### Reliability and validity of the findings

The study pre-tested the research protocols in one selected deaf institution, namely ‘deaf Action, Uganda under Immanuel church for the deaf in Makerere Kikoni, Kampala’. This process helped to identify issues like the use of charts and pictures for clear explanations. It further helped to determine how this group would respond to the study design and research questions, which reduced the challenges, errors, risks and reliability in the subsequent study. Furthermore, data triangulation was used in this study to strengthen the validity of the findings. For instance, this study used three methods of data collection (interviews, FGDs and document analysis). During this process, the study compared results from different sources to check the consistency of the findings.

My reflexivity stems from being a UGSL interpreter who holds a diploma in UGSL from Kyambogo University and a certificate in UGSL from the Centre for Sign Language Bible Translation Network Uganda, under the UNAD. My experience as a sign language interpreter conducting research among deaf women was halfway easy because my educational background and experience with deaf people helped to identify as a member of this group. Being able to use sign language helped to build trust with deaf women and girls, I was considered an insider, and this position did not affect the study because I was aware of possible biases. However, I always reflected on the objectives and triangulation of different methods, which I used to avoid biases. However, the uniqueness of researching the experiences of deaf people exhibits some challenges. These included slightly different sign language, especially among uneducated deaf women who used home signs; some participants narrated long stories or lengthy personal accounts that were irrelevant to the topic, but still we had to listen as professional sign language interpreters and video-record all the activities.

### Limitations

There is an absence of disaggregated data on deaf women and girls or situational analysis on their MHRs. This challenge was identified when I visited the key selected government ministries; then I requested government documents or analysis reports on deaf women and girls on MHRs and services in Uganda. Shockingly, I was informed that they did not have any detailed information about deaf women or girls; rather they had some information about PWDs as a group. To mitigate this challenge, I administered unstructured interviews with key informants with the view of getting detailed understanding and expert knowledge on the matter. However, a healthy environment that facilitates the inclusion of deaf women and girls is urgently needed.

Additionally, Mbale School for the deaf was and still is under investigation over corruption allegations involving school administrators manipulating student enrolment figures to secure increased funding, among other allegations. Therefore, my team members were reduced, and questions relating to the population of the students were not shared. Moreover, some members of staff became sick, while others had emergencies. Luckily, I was able to get interviews from the students and some key informants from the school. However, the research team mitigated all these challenges by patiently following all the instructions that were offered to us.

### Ethical considerations

The study was approved by the Makerere University School of Public Health Institutional Review Board (MAKSHSREC-2023-477) and registered at Uganda National Council for Science and Technology Number (UNCST-SS2222ES). This study involved talking to deaf women and girls and other stakeholders on MHRs and services. I was aware that talking to this group of people about their lived experiences might evoke emotional stress and feelings of helplessness and cause unnecessary distress. Therefore, the following ethical action steps were taken: I ensured that all study materials that relate to maternal health services at the community level (including the consent forms and charts) were translated from English to sign language (by signing, facial expression and body movements).

Notably, there were serious concerns about informed consent in sign language that should be written, especially during the Review Board (School of Health Science). But Lutalo-Kiingi (2014) explains that sign language is a natural language where people communicate using hands and bodily gestures instead of their mouths, primarily used by people who are deaf, hard of hearing or have hearing difficulty or are unable to vocalise speech and sign language interpreters.

## Results

This study was undertaken to understand the MHRs and services of the deaf women and girls in Uganda, specifically those who are profoundly deaf or hard of hearing. In order to find ways of improving access to these rights and services, the following questions emerged: (1) *What maternal health rights and services, awareness and practices do deaf women and girls have?* (2) *What are your sources of information about maternal health rights and services?* (3) *What contributions has the government as a duty bearer made towards implementing maternal health rights and services for deaf women and girls?*

The findings from the study revealed a wide range of factors that limited access to MHRs and services for the deaf women. For example, focus group findings revealed that deaf women and girls faced several challenges when accessing MHRs and services. A total of 50 participants were interviewed, yielding a response rate of 99%. This is because the participants understood and responded adequately to all the questions to inform the objectives (see Table 1 for background information of the participants).

**TABLE 1:** Background information of the participants (*N* = 63).

Position of participants	Data collection method	Number of participants
Deaf women (25); Adolescent deaf girls (25)	Focus group discussions and follow-up in-depth interviews	50
Executive officer from Uganda National Association of the Deaf (UNAD)	Key informant interviews	1
Medical officers (2); Village Health Team (VHT) (2)	Key informant interviews	4
School administrator (1); Sign language interpreters (2)	Key informant interviews	3
Deaf husband	Key informant interviews	1
Executive officers from the Ministry of Health (1); Ministry of Gender (1); Ministry of Education and Sports (1)	Key informant interviews	3
Community Development Officer (CDO)	Key informant interviews	1

### What maternal health rights and services, awareness and practices do deaf women and girls have?

#### Communication barriers

All 50 participants attested to the fact that they were fully aware of the need for antenatal care for pregnant women and girls. Also, they understood the importance of attending antenatal clinics, but their perception of the activities during the antenatal visits differed and was misunderstood because of communication barriers. Ten participants used the method of writing on paper for explanations, but they indicated that the process of writing was slow and English words were hard to write, which made communication challenging for them, especially during labour and childbirth. Moreover, some participants did not like visiting the hospital with their family members, as it deprived them of their right to privacy and confidentiality. They believed that family members could gossip about the whole family:

‘I know that all pregnant women usually go to the hospital for check-ups and advice to ascertain the health status of the baby, unfortunately, such advice is adversatively directed to hearing women, but for deaf women, we don’t have sign language interpreters to explain what is going on that is why I don’t go for antenatal visits.’ (Participant 2; 37 years old; FGD 5)

The deaf husband stated:

‘My wife is uneducated, but she simple SL, when we go to the hospital, I communicate to the nurses by writing down. She cannot go to the hospital alone due to communication barrier.’ (Participant 12; 56 years old; In-depth)

#### Lack of antenatal information and classes

Some deaf women indicated that they had visited health facilities for maternal health services, but a lack of antenatal information and classes targeting deaf women affected their ability to be aware of such services; for example, 20 deaf women reviewed that they were ignorant and unaware of the consequences of not having tested for HIV and AIDS during pregnancy, and such information about mother-to-child transmission is not tailored and available for deaf women in the health centres. These women indicated that such information is missing in their reproductive lives, and there are no tips or advice on how to care for their newborn babies, which is so risky for the baby’s health:

‘When I was pregnant my friend told me how she was informed that she infected her newborn baby with HIV [*and*] AIDS, after giving birth. She didn’t know that she had HIV [*and*] AIDS, then suddenly, her baby became sick, then they carried out different tests, and she was told so. You see, we are never informed of all these critical issues. I’m also ignorant and unaware of the consequences of not having tested for HIV [*and*] AIDS during pregnancy. There are no counselling sessions for such deaf mothers with HIV [*and*] AIDS and also a lack of awareness information about mother-to-child transmission tailored and available for deaf women in the health centres. But these services are available to hearing women.’ (Participant 3; 36 years old; FGD 4)

Shockingly, many of these deaf women and girls did not know about mother-to-child transmission and how HIV and AIDS can infect the unborn baby. Some of the deaf women shared that they could not afford the price of testing for HIV and AIDS, which further complicates their lives. Although some of them received these services, they did not get any advice on the outcome of the tests, which made them ignore or avoid such tests. One participant revealed that she had been receiving few antenatal services in the health centre and Mbale Referral Hospital, but both health centre and hospital did not have sign language interpreters or services for deaf pregnant women to explain why she needed the tests and the medications.

#### Negative attitude by the medical officers

At the deaf school, girls who had experiences with maternal health care revealed negative attitudes from the health workers made it hard for the pregnant adolescents to access services in the health centre. These health workers could show a rude facial expression and questions, which made these teenagers to avoid antenatal services. A participant shared as follows:

‘Two years ago, I became pregnant then I went to the health centre for advice. Instead I was treated so badly. I cannot tell what she was saying but basing on her facial expression and pointing to my stomach. I realised that it was a mistake approaching this place. So I panicked and run away.’ (Participant 4; 17 years old; In-depth)

The school administrator shared as follows:

‘[*S*]ome girls became pregnant and gave birth at a very young age due to a lack of sex education. The challenge is that teenage pregnancies also affected the deaf girls in this district, but I was not investigated. I think government should also consider including deaf related programmes especially on maternal health services, because they are left out.’ (Participant 6; 48 years old; Key)

Moreover, the executive from the UNAD also shared that there are no maternal health policies that have included the linguistic needs of deaf women and girls, which have totally excluded the deaf. Similarly, the CDO revealed that (Gimono 2025:123):

[*T*]here are no maternal health programmes at the district and local councils that target the deaf women in the communities apart from the general programmes that engage all PWDs at the community level. (Participant 7; 42 years old; Key)

He revealed how he had never thought of how they access health services without sign language interpreters and wondered how they get the required services. When I further interviewed the government departments, they revealed that there is no specific law or policies enacted to cater for the deaf community’s needs, except for the *Persons with Disabilities Act 2020*, which combines and generalises all the interests of all PWDs in Uganda.

#### Sign language interpreters

All participants and health workers shared that there are no sign language interpreters in the health centre, which makes it difficult for the deaf women to communicate with the health workers. A health worker noted:

‘It is true, we do not have SL interpreters in this health centre which make our work difficult to work on the deaf people. I think government through ministry of health should think about deaf pregnant women and girls.’ (Participant 8; 46 years old; Key)

One deaf woman noted:

‘I was given the medicine when I was pregnant, but there were no explanations on how and when to take the medicine. I just took it by guessing. We need SL interpreters to help us understand what the nurse is explaining to avoid overdose which can lead to miscarriage.’ (Participant 5; 29 years old; In-depth)

These findings show that a lack of antenatal care and medication instructions for deaf women can lead to significantly increased risks of complications during pregnancy and childbirth, which can lead to maternal mortality and infant mortality.

### What are the sources of information about maternal health rights and services?

Focus group discussion participants and interviewees identified deaf friends, siblings and some health workers as the main sources of information on maternal health services. However, most adolescents, especially those in school, relied mostly on their deaf peers and grandparents for information on maternal health. (Source of information on antenatal and postnatal services among deaf women and girls; see Table 2.)

**TABLE 2:** Source of information on antenatal and post-natal services among deaf women and girls.

Source of information	Deaf women	Deaf girls	*n*	%
Friends and family members	25	15	40	80
Health centre	-	-	-	-
Did not have any source of information on maternal health rights and services	-	10	10	20

**Total**	**25**	**25**	**50**	**100**

Out of 50 participants, 40 participants reported friends and family members as their source of information about antenatal and postnatal care. A total of 10 participants did not have any source of information because they were teenage mothers who did not seek any advice because of fear of being thrown out of their parents’ homes. Among these women and girls, 25 women confirmed family and friends were their source of information, while 15 girls also confirmed the same:

‘Since there was no detailed information on antenatal care and even after birth. I only rely on my husband. The problem is my husband is also deaf, but he can write. You see this process is so slow especially when I was ready to give birth. I could not wait. As my husband was writing down, I was busy pushing the baby on the floor. The nurses just look at us waiting to see, what he is writing or walk away. But I go with my husband.’ (Participant 1; 33 years old; In-depth)

‘My fellow friends with children advised me, when I’m pregnant.’ (Participant 4; 23 years old; FGD 3)

All participants suggested that the government should employ sign language interpreters in all health centres and hospitals in Uganda, especially female sign language interpreters in all health centres, to work on sensitive issues of maternal health for deaf women and girls. It is vital to improve communication accessibility between the health providers and promote deaf epistemology (deaf way of thinking) in the health sector to improve better service delivery.

### What contributions has the government as a duty bearer made towards implementing maternal health rights and services of deaf women and girls?

This question was set to determine the contributions made by the government of Uganda in meeting its duties as a state party to the international human rights frameworks, standards and principles, and how duty bearers’ performance deviates from the ideal norm. Therefore, any revealed gaps may need to be resolved before rights can be realised and accessed, and it is important for the identification of areas in need of contribution from duty bearers to address the various challenges of accessibility and services. This objective was achieved through the process of interviewing key informants from government officers and other stakeholders working in the deaf community, including the Ministry of Health; Ministry of Education and Sports; Ministry of Gender, Labour and Social Development; Deaf school; UNAD and CDO.

The major challenge reported was limited funds allocated to these ministries to promote and create awareness of maternal health programmes among deaf people. Moreover, it was further pointed out that because of a lack of empirical research on deaf women’s reproductive health care, it is hard to plan for such a group on such issues. As affirmed by the executive officers. The Ministry of Health executive officer stated:

‘We are allocated limited funds which are directed to do specific programmes. Remember, these funds are given based on the activities forwarded by each ministry. So creating awareness is done in the general and universally acceptable methods. We do not provide special care for the deaf people.’ (Participant 9; aged 54 years old; Key)

The UNAD executive officer stated:

‘There is an urgent need to conduct empirical studies on maternal mortality results and teenage pregnancies among deaf women and girls. Currently, there is no research speaking about the voices of these women on their maternal health rights and services.’ (Participant 10; 48 years old; Key)

The Ministry of Gender executive officer stated:

‘There are no clear statistics on the population of deaf people. Most of the strategies and policies we make, generalise all those people in one group as “Persons with Disabilities, PWDs.”’ (Participant 11; 46 years old; Key)

These findings indicate that deaf girls need a lot of care and support from the government, counsellors, interpreters, parents, teachers and the community. One could, therefore, be optimistic and conclude that with the support of the government, counsellors, UGSL interpreters, teachers, parents and the community, adolescents would access the information they need to protect themselves from sexual and reproductive health (SRH) problems. Once informed, adolescents too can play an important role in passing on the information to their peers. This will potentially help in reducing the number of unintended teenage pregnancies that have increased, according to the current UNICEF (2019), UBOS (2016) and United Nations Population Fund (UNFPA) statistics of 2012 (UBOS & ICF 2018), AIDS and Sexually Transmitted Infection (STI) incidence among adolescents in Uganda.

This chapter has shown the influences on accessing maternal health care and services for deaf women and girls. Communication barriers, negative attitudes, lack of maternal information tailored for deaf women and lack of sign language were identified as influences that prevented deaf women from accessing maternal health services. Among sources of maternal information, it was revealed that relatives and friends provided critical support in creating awareness of maternal health issues.

## Discussion

In this study, satisfaction and accessibility of MHRs and services, especially antenatal care and clinic services among deaf women, were relatively low. The findings revealed deaf women’s dissatisfaction with maternal health care and services that were associated with lack of accessible antenatal information in sign language, communication barriers, lack of SL interpreters and negative attitudes by the health workers towards the deaf pregnant women. These findings are consistent with previous studies, which showed that the would-be mothers were dissatisfied with the antenatal services rendered by physicians, midwives and nurses and other allied health care workers when compared with the hearing pregnant women who attended the same clinics for antenatal care (Adigun & Mngomezulu 2020; O’Hearn 2006). As noted by Adigun and Mngomezulu (2020), ineffective two-way communication and the interaction between deaf patients and the midwives and/or their physicians had negatively impacted their experiences at the clinics. Mitra et al. (2020) revealed that deaf women frequently struggle with healthcare communication, and this may be a potential driver for some of the disparities that were identified.

Moreover, awareness and access to information about maternal health services are one important component of decision-making in reproductive health (Mitra et al. 2020). Women’s awareness of their right to access maternal health information in accessible formats contributes to empowerment and the ability to make informed choices regarding the utilisation of available services (Adigun & Mngomezulu 2020). The findings of the study also show a low level of awareness of strategies and accessible formats about the right to access maternal health services among deaf women because of inadequate interpretation services in the health centres. In addition, inadequate interpretation of maternal health services among deaf women and girls has previously been cited as one of the challenges (Adigun & Mngomezulu 2020; Gichane et al. 2017; Mitra et al. 2020), yet the ability of deaf women and girls to assert their rights and demand services from their society is dependent on their capacity to communicate effectively with others (Mitra et al. 2020). The current study showed that the communication barrier between deaf women and health workers still exists. In my view, these deaf women tried to seek antenatal services from the health centres but were not aware of the communication challenge and how it would impact their pregnancy and childbirth. These findings call for the application of the HRBA principles of accessibility, acceptability, availability and quality to identify and monitor all Sexual and Reproductive Health Rights (SRHR) practices from the perspective of access and equality for deaf women and girls (UNFPA 2019; WHO 2012).

Indeed, the current study showed that deaf pregnant women seeking antenatal and postnatal care and childbirth failed to understand the medical officers when accessing maternal health information, medical instructions during childbirth and the prescribed antenatal medication because of inadequate communication modes that are not in sign language. As noted in previous research, much of the information given during antenatal visits is largely presented verbally, which deprives deaf women of essential antenatal information (Adigun & Mngomezulu 2020). It is, therefore, assumed that deaf women and adolescent girls are likely to be marginalised and excluded from accessing adequate reproductive health information from midwives and other health practitioners because communication modes are not in sign language (Human Rights Watch 2010; Adigun & Mngomezulu 2020).

However, this study also found that deaf women struggled to understand the importance of seeking antenatal services during pregnancy and delivery because of the lack of special corners that meet their needs. The silence and stigma around maternal health care issues might prevent deaf women from articulating their needs, let alone seeking services. This omission may be one of the factors that prevented these women from accessing these services and forced them to stay at their homes until childbirth. Certainly, this predisposes these deaf pregnant mothers and girls to life-threatening conditions, including loss of lives. One of the major concerns of the social model of disability is to eliminate social barriers that limit deaf women from active participation and inclusion in maternal rights and care. This model emphasises the need to uphold maternal rights and ensure proper and effective services required by the deaf community, such as the availability of sign language interpreters during the antenatal sessions in the health centres. Shockingly, it was revealed that deaf pregnant women rarely go for HIV and AIDS testing because they did not know its importance, and those who had HIV and AIDS did not know where to access the medication and counselling. Such social barriers can intensify the risk of pregnancy complications and mother-to-child transmission.

Shakespeare (2010) argues that the social model is a ‘practical tool, not a theory, an idea or a concept’; rather, it demonstrates that the problems disabled people face are the result of social oppression and exclusion, not their deficits. This places the moral responsibility on society to remove the burdens that have been imposed and to enable disabled people to participate (Shakespeare 2010). It is further reflected that the state has the responsibility to address socially created obstacles to ensure full respect for the dignity and equal rights of all persons. Although this approach calls for inclusive societies to ‘value difference and respects the dignity and equality of all human beings regardless of difference’ (Shakespeare 2010), there is still more need to be done to fulfil these objectives. As already shown in this study, the principles of social model of disability in the lives of the deaf community and spaces are yet to be fully realised and achieved.

It was revealed that all policies and strategies on MHRs are made for everyone in Uganda (one-size-fits-all). It was further voiced that there are no special needs policies or strategies and programmes that are made for deaf people. This shows that the government of Uganda has neglected its international and regional human rights obligations. The absence of targeted policies and programmes towards deaf individuals can lead to increased societal stigma and discrimination surrounding their disability and sexuality. It is therefore necessary for the government to design laws and policies that do not contradict international human rights obligations.

The absence of deaf epistemology in policy-making processes, research and maternal health education means that their specific needs and rights may not be adequately addressed. It is through the deaf way of knowing their lived experiences, stories and advice that effective and appropriate laws, policies, research and programmes will meet the needs of the deaf people in Uganda.

Inadequate SL interpretation of maternal health services among deaf women has previously been cited as one of the challenges (Gichane et al. 2017; Adigun et al. 2021); yet the ability of deaf women and girls to assert their rights and demand services from their society is dependent on their capacity to communicate effectively with others (Adigun et al. 2021). The current study showed that the communication barrier between deaf women and health workers still exists. In my view, these deaf women tried to seek antenatal services from the health centres but were not aware of the communication challenge and how it would impact their pregnancy and childbirth. These findings call for government as the duty-bearer to apply the HRBA to promote participation and empowerment of these women to claim their rights and actively engage in their maternal health decision-making processes (UNFPA 2019; WHO 2012).

The HRBA contains four key human rights-based preconditions needed for SRHR: AAAQ (UNFPA 2019). This means that healthcare facilities, goods and services must be sufficiently and continuously available for all people who need these services. Although the human rights-based principles require that these services need to be affordable and accessible for all in a non-discriminatory manner and culturally appropriate and sensitive to the issues of gender, age, disability and other characteristics (UNDP 2006), the current study shows that there is more that needs to be done in fulfilling these needs and standards. From the human rights point of view, it could be concluded that denying access to maternal health services, HIV testing, lack of participation and inclusion of deaf women and girls in their antenatal and childbirth processes is a violation of their reproductive rights as provided in the Ugandan Constitution, 1995, under article 33(3), which reveals that ‘the state shall protect women and their rights, taking into account their unique status and natural maternal functions in society’.

This study shows that the human rights standards of AAAQ of antenatal and postnatal care among this group are perceived to be lacking in the Ugandan health sector. For instance, while the WHO recommends at least four ante-natal visits during pregnancy and that post-natal care must be in place within 2 days of the birth, deaf mothers and adolescent mothers reported several instances where they did not access ante-natal and postnatal services. This could be attributed to a lack of awareness programmes, communication barriers, a lack of sign language interpreters and the unavailability of skilled personnel in health facilities to guarantee safe delivery to expectant deaf women and girls.

In summary, the HRBA reveals that the right of every girl and woman to health is not only about providing technical health care and services but also entails girls’ and women’s empowerment and enabling them to have the freedoms and entitlements. They need to exercise autonomy and agency over their lives and bodies and decide freely and responsibly on all matters directly or indirectly related to their health, free from violence and discrimination.

In light of the findings of this study, more effort is needed to promote deaf epistemology. It is through the ‘deaf way of knowing’ that evidence-based strategies will be made to improve maternal services among deaf women and girls. Therefore, deaf epistemology is an opportunity for society to understand clearly ‘deaf ways of being in the world, of conceiving that world and their place within it, both in actuality and in potentiality’. This paradigm should be considered as a valuable tool in developing policies and practices aimed at improved quality of maternal health among this group.

### Policy and programmatic implications

The findings of the study have important implications for policy-making and programme design. Deaf women and girls use sign language as the only way of communication, which is often overlooked in maternal health policy-making, programme designing and service delivery. The overlook of these needs has created drastic barriers and hindered accessibility to maternal health services for this group. Therefore, there is a need to include SL interpreters in the public service commission so that they are fully posted in each health centre, just like the medical officers for speedy and smooth service delivery.

### Recommendations

Deaf women and girls suggested that the government should adopt innovative means of dissemination of maternal health information that is tailored to their linguistic needs. This is because deaf people listen with their eyes. Therefore, visual materials are the most useful tools to communicate and receive information. For instance, posters together with drawings and displays of maternal pictures in all health centres; drama plays at community levels, health centres and places of worship and the use of dolls as awareness materials for demonstration in antenatal classes at health centres.

Moreover, efforts to promote maternal health among deaf women require the government to integrate human rights principles in different phases of the programming, including assessment and analysis, programme planning and design, implementation, monitoring and evaluation. Human rights-based approach offers a mechanism which ensures that rights-holders claim their rights while duty-bearers fulfil their obligations to realise these rights. In policy terms, the government should also integrate the social model of disability in all its programmes, strategies and policies because it is an essential model that calls for equal opportunities, the elimination of barriers that limit PWDs from participating and decision-making in their reproductive rights.

## Conclusion

The study findings suggest that serious challenges lie ahead in the provision of safe motherhood information for deaf women and girls in Uganda. Despite Uganda’s legal frameworks on MHRs, deaf women and girls’ linguistic needs are yet to be incorporated into the Ugandan health sector. Current healthcare provisions do not always meet their needs during maternal services. Therefore, visible and constructive policies are necessary to steer deaf MHRs and services. In other words, further research is needed to understand HIV and AIDS awareness, Antiretro-viral drugs (ARV) and counselling for services to deaf pregnant women. This can increase HIV and AIDS awareness, advocacy and support for deaf pregnant women.
